# COXPRESdb v7: a gene coexpression database for 11 animal species supported by 23 coexpression platforms for technical evaluation and evolutionary inference

**DOI:** 10.1093/nar/gky1155

**Published:** 2018-11-20

**Authors:** Takeshi Obayashi, Yuki Kagaya, Yuichi Aoki, Shu Tadaka, Kengo Kinoshita

**Affiliations:** 1 Graduate School of Information Sciences, Tohoku University, 6-3-09, Aramaki-Aza-Aoba, Aoba-ku, Sendai 980-8679, Japan; 2 Tohoku Medical Megabank Organization, Tohoku University, Sendai 980-8573, Japan; 3 Institute of Development, Aging, and Cancer, Tohoku University, Sendai 980-8575, Japan

## Abstract

The advent of RNA-sequencing and microarray technologies has led to rapid growth of transcriptome data generated for a wide range of organisms, under various cellular, organ and individual conditions. Since the number of possible combinations of intercellular and extracellular conditions is almost unlimited, cataloging all transcriptome conditions would be an immeasurable challenge. Gene coexpression refers to the similarity of gene expression patterns under various conditions, such as disease states, tissue types, and developmental stages. Since the quality of gene coexpression data depends on the quality and quantity of transcriptome data, timely usage of the growing data is key to promoting individual research in molecular biology. COXPRESdb (http://coxpresdb.jp) is a database providing coexpression information for 11 animal species. One characteristic feature of COXPRESdb is its ability to compare multiple coexpression data derived from different transcriptomics technologies and different species, which strongly reduces false positive relationships in individual gene coexpression data. Here, we summarized the current version of this database, including 23 coexpression platforms with the highest-level quality till date. Using various functionalities in COXPRESdb, the new coexpression data would support a broader area of research from molecular biology to medical sciences.

## INTRODUCTION

Owing to high-throughput technologies, a huge volume and variety of data is currently available in public repositories. RNA-sequencing (RNAseq) technologies have been increasingly used in recent years, while microarray technologies are also being widely used for basic transcriptomics experiments. Both technologies have resulted in the perpetual growth of transcriptome data generated under various cellular, organ and individual conditions in a wide range of species. However, cataloging all transcriptome conditions would be a mammoth task, considering the sheer number of intercellular and extracellular conditions. Timely use of such growing data is key to promoting relevant research in molecular biology.

Gene coexpression relationship is relationships of genes with similar expression profiles in large amount of transcriptome data. Considering the strong association between gene expression and its function, also known as guilt-by-association, gene coexpression information can provide an accurate prediction of gene function. Importantly, the quality of coexpression data strongly depends on the sample size (1,2). Larger number of samples results in more effective discrimination of subtle but substantial differences in the cellular context, hence providing a precise clue to the biological function of each gene.

To promote the usage of gene coexpression information, many gene coexpression databases have been made available, especially in plant science (see reviews; 3–6). We have also developed a coexpression database for animal researches. COXPRESdb (COeXPRESsed gene DataBase; http://coxpresdb.jp) was first released for human and mouse in 2007 (7). Through periodic updates, we have developed functionalities to enhance usability of gene coexpression information; for example, searching coexpressed genes using functionally related multiple query genes (7), drawing coexpressed gene network with pathway and protein–protein interaction information (8), and automatically detecting and analyzing submodule structures of coexpressed gene networks (9). Furthermore, we have expanded the target species and platforms, and have continued the development of coexpression calculations and quality assessment methodologies (10). One important point about coexpression calculation is that the coexpression relationship is a summary of a given set of transcriptome data and thus the quality of coexpression data strongly depends on that of the underlying transcriptome data. Importantly, every transcriptome data intrinsically includes some bias from technical and biological viewpoint; different technology has different systematic noises and particular species are preferentially selected for particular research topics. To infer less biased coexpression relationships, comparison of independent coexpression data is effective. One characteristic feature of COXPRESdb is the provision to compare multiple coexpression data derived by different transcriptomics technologies and from different species (8,9). Moreover, interspecies comparison can provide insight for lineage-specific coexpression evolution (11,12). The key to enhance the relevance of intra- and interspecies comparison of coexpression data is the quality and quantity of coexpression data, and in COXPRESdb version 7 this has been largely improved. Through various functionalities in COXPRESdb version 7, the new coexpression data can strongly support a broader area of research from molecular biology to medical sciences.

## OVERVIEW OF THE LATEST COEXPRESSION DATA

### New coexpression data

In addition to the update of all the 15 coexpression platforms previously provided in COXPRESdb, we have added eight new RNAseq-based coexpression platforms for nematode (Cel-r), dog (Cfa-r), zebrafish (Dre-r), chicken (Gga-r), monkey (Mcc-r), rat (Rno-r), budding yeast (Sce-r) and fission yeast (Spo-r) (Table 1). Therefore, multiple coexpression platforms are now made available for all the 11 species in COXPRESdb. To retrieve condition-independent coexpression information from a given gene expression matrix, sample redundancy should be reduced. However, definition of redundancy of sample condition is not easy. In addition to fully redundant experiments, there are many similar tissues and cellular conditions. For this problem, we previously adopted an approach of weighted correlation coefficient based on a computationally calculated redundancy of each sample (7). However, this method enhanced not only the worth of valuable samples for minor conditions but that of samples just having noisy measurements. To solve this problem, in COXPRESdb version 7, we adopted principal component analysis as a dimension reduction technique of partially or fully redundant samples. Since each principal component reflects a biological factor (13), the principal component space can be used as a less biased sample space. Combined with random sampling technique of samples/conditions (2), condition-independent coexpression information was prepared. In the following sections, we describe three types of summaries to evaluate the quality of coexpression data from different aspects.

**Table 1.** The latest coexpression dataset provided in COXPRESdb

Species	Coexpression platform ID	Version	Transcriptome platform	Genes	Samples
*Caenorhabditis elegans*	Cel-m	c4-0	A-AFFY-60	17256	1780
*Canis lupus*	Cfa-m	c3-0	A-AFFY-149	16214	777
*Drosophila melanogaster*	Dme-m	c4-0	A-AFFY-35	12626	4209
*Danio rerio*	Dre-m	c4-0	A-AFFY-38	10112	1423
*Gallus gallus*	Gga-m	c4-0	A-AFFY-301	13757	1502
*Homo sapiens*	Hsa-m	c5-0	A-AFFY-44	20283	14347
*Homo sapiens*	Hsa-m2	c3-0	A-AFFY-141	20199	20199
*Macaca mulatta*	Mcc-m	c3-0	A-AFFY-145	15782	1006
*Mus musculus*	Mmu-m	c4-0	A-AFFY-45	20962	20962
*Rattus norvegicus*	Rno-m	c4-0	A-AFFY-43	13751	13751
*Saccharomyces cerevisiae*	Sce-m	c3-0	A-AFFY-47	4461	3593
*Schizosaccharomyces pombe*	Spo-m	c3-0	A-AFFY-47	4881	166
*Caenorhabditis elegans*	Cel-r	c1-0	Illumina	13690	1546
*Canis lupus*	Cfa-r	c1-0	Illumina	15303	253
*Drosophila melanogaster*	Dme-r	c2-0	Illumina	11937	4596
*Danio rerio*	Dre-r	c1-0	Illumina	18446	3049
*Gallus gallus*	Gga-r	c1-0	Illumina	15554	698
*Homo sapiens*	Hsa-r	c2-0	Illumina	17067	10485
*Macaca mulatta*	Mcc-r	c1-0	Illumina	15050	1205
*Mus musculus*	Mmu-r	c2-0	Illumina	17095	7278
*Rattus norvegicus*	Rno-r	c1-0	Illumina	15410	2368
*Saccharomyces cerevisiae*	Sce-r	c1-0	Illumina	5674	1205
*Schizosaccharomyces pombe*	Spo-r	c1-0	Illumina	5310	143

### Quality assessment of coexpression data by pathway annotations

**Figure 1.**
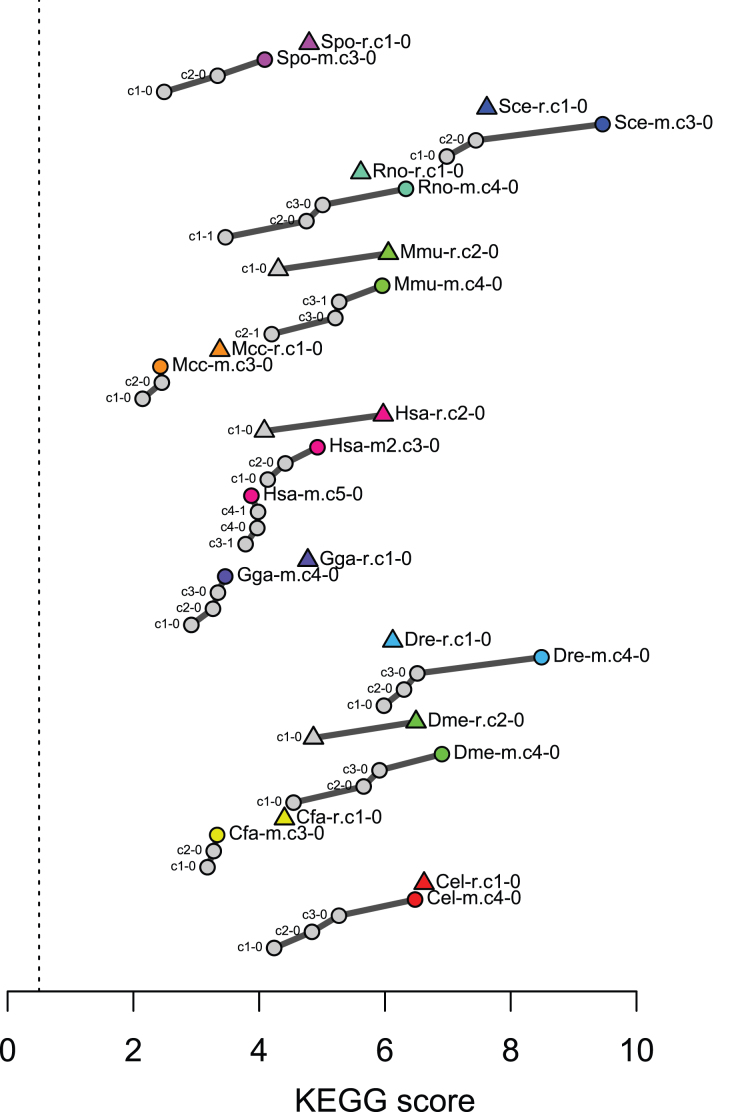
Progression of KEGG score of the coexpression data in COXPRESdb. The KEGG score shows the consistency of coexpression data with KEGG pathway annotation for each species. The following number of KEGG pathways associated with less than 50 genes were used for this quality assessment; 104 for Cel, 127 for Cfa, 97 for Dme, 86 for Dre, 98 for Gga, 117 for Hsa, 117 for Mcc, 111 for Mmu, 108 for Rno, 95 for Sce and 93 for Spo. The scores of the current coexpression version are also shown in Figure 3.

First, we checked the consistency of coexpression data with pathway annotations. Since coexpression information is used as an estimator of co-function relationships, genes in the same pathway are expected to show strong coexpression. For gene function, in relation to gene coexpression, we used KEGG pathway annotations (downloaded on 28 February 2018) (14), which cover a broad range of species with similar annotation density, enabling not only comparison of coexpression data quality within a species but also allowing a rough comparison across different species. We selected highly specific KEGG pathways that were associated with <50 genes in each species, resulting in 104.8 pathways on average for the 11 species (standard deviation = 12.4). Using these KEGG pathway annotations, we tested whether a coexpressed gene pair has at least one common KEGG pathway annotation or not. This discrimination performance, which is hereafter referred to as *KEGG score*, was quantified by a partial area under ROC curve (false positive rate = 1%), as described previously (2), with slight modification in ROC calculation using a weighting by the inverse of the number of genes in each pathway, namely a weighted ROC curve. This modification gives a larger weight for more specific pathway annotations, resulting in a robust assessment against selection of a threshold of the number of genes in a pathway (50 in this report). The KEGG scores for the previous and current coexpression data in COXPRESdb are shown in Figure 1, indicating generally continuous improvements of the coexpression data. These improvements are mostly as a result of increased number of publicly available transcriptome data as well as revisions of coexpression calculation methods. It would be worth noting that we did not use all the available samples for Hsa-m platform owing to the high calculation cost. The Hsa-m platform, along with the other platforms that did not show substantial improvement, would require further methodological development.

### Similarity among coexpression platforms

**Figure 2.**
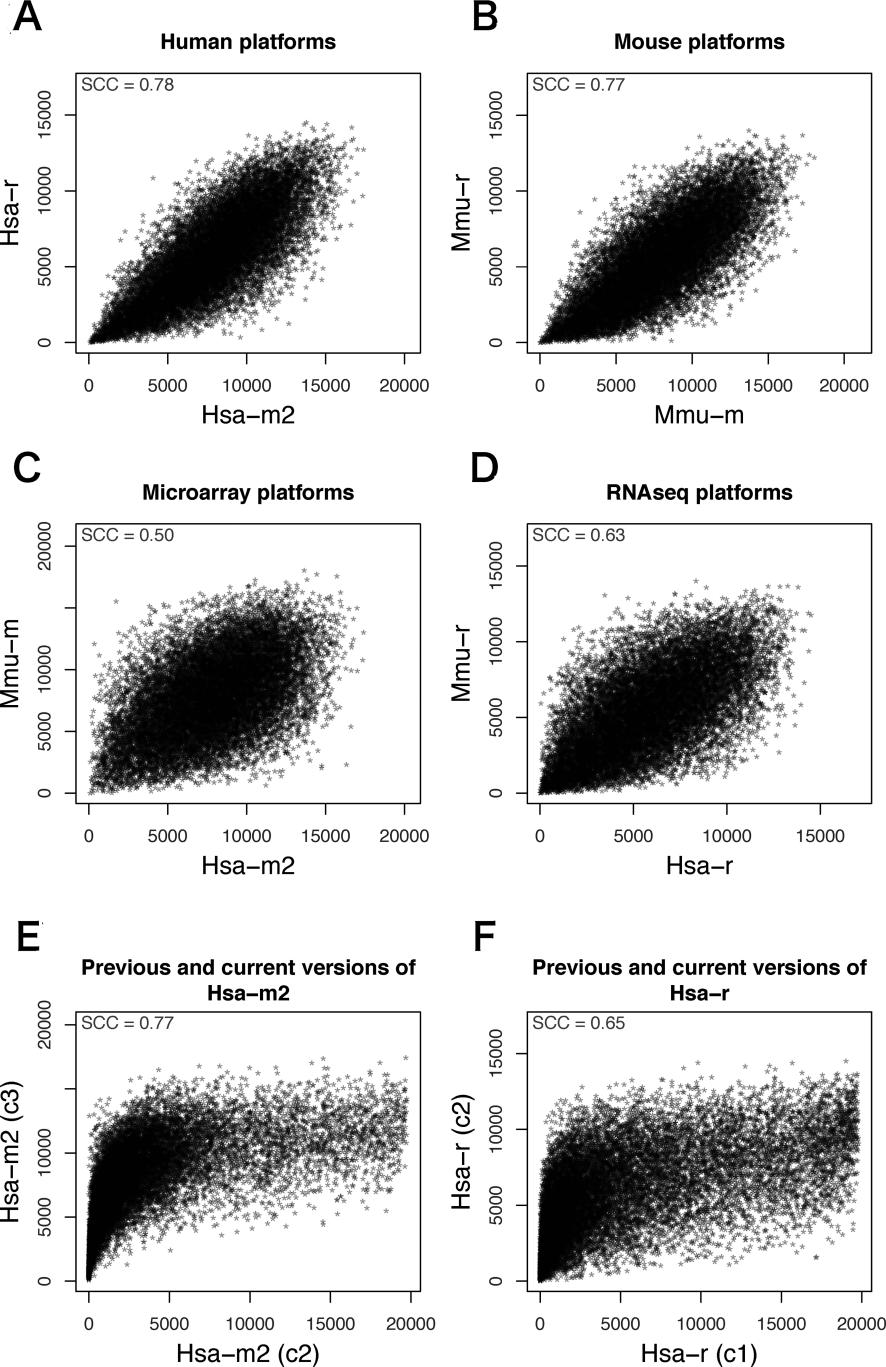
Comparison of MR values in the four platforms in human and mouse. MR index values in different platforms/versions have been plotted, where smaller value indicates stronger coexpression. Intraspecies difference (**A**, **B**) is smaller than interspecies difference (**C**, **D**). Difference between RNAseq platforms is smaller than that between microarray platforms (C, D). Distribution of MR values in the current version are different from that of the previous version (**E**, **F**). The details of the coexpression platforms are shown in Table 1. SCC; Spearman's rank correlation coefficient.

Comparison of multiple coexpression data is the central idea for evaluating and improving the reliability of coexpression data. First, we compared four coexpression platforms (Hsa-m2, Hsa-r, Mmu-m and Mmu-r) using 16 110 gene pairs, which commonly appear in any coexpression platform in COXPRESdb. The MR index, which is the measure of coexpression strength in COXPRESdb (15), showed good correspondence between the two platforms for human and mouse, respectively (Figure 2A and B). Note that since smaller MR value indicates stronger coexpression, intersect of data in the lower-left area of the graph represents strong functional prediction. In contrast, data points in the upper-right area of the graph are indicative of coexpression having an anti-correlation relationship, which is not apparent in the current coexpression data in COXPRESdb. Interspecies differences (Figure 2C and D) were larger than intraspecies differences (Figure 2A and B), as expected. Interestingly, the interspecies difference on RNAseq platforms (Figure 2D) was smaller than that on microarray platforms (Figure 2C), thereby suggesting a lower technical bias in the RNAseq platforms, although the number of samples in RNAseq platforms are generally smaller than those in microarray platforms. Please note that the distribution of MR values in the current version is different from that in the previous versions due to the modification of coexpression calculation method. In the previous versions, MR values are almost uniquely distributed because MR index is derived from an order index. In the current version, we adopted a methodology using random sampling and aggregation for MR calculation, resulting in a skew normal distribution of the MR values (Figure 2E and F).

**Figure 3.**
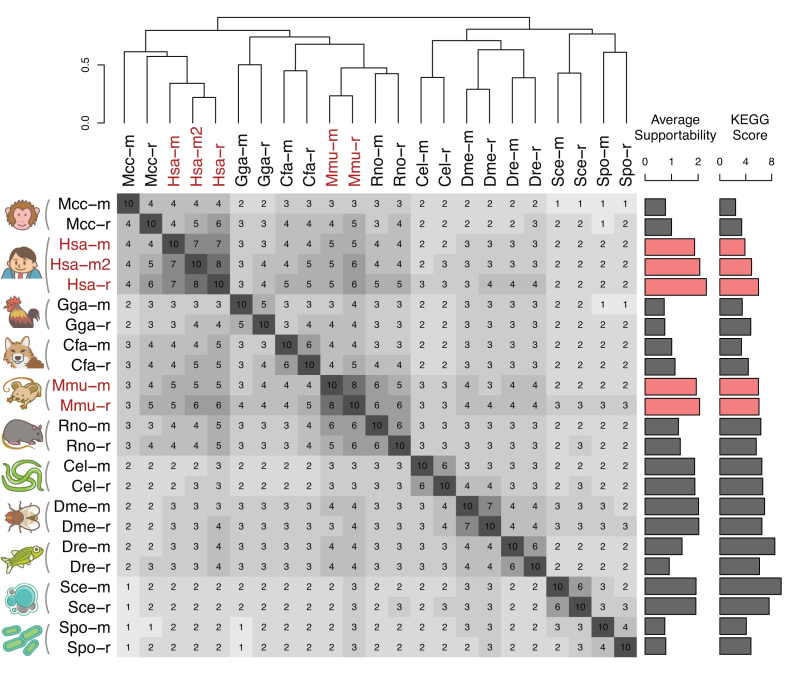
Similarity among the current coexpression data in COXPRESdb. Coexpression similarity was calculated using Spearman correlation coefficient for the coexpression values for 16 110 gene pairs among the 180 one-to-one orthologous gene groups. The 180 orthologous genes have expression values for all the 23 platforms provided in COXPRESdb. For visualization of the correlation matrix, 10-fold values of the Spearman's rank correlation are displayed. The correlation matrix was hierarchically clustered by the complete linkage method for correlation distance (1 – correlation). KEGG scores in Figure 1 and average supportability calculated from Figure 4 are also presented as bar plot. Platforms for human and mouse are highlighted, which show high reproducibility.

To grasp global relationships among the 23 coexpression platforms (Table 1), a similarity matrix of the platforms was constructed using Spearman's rank correlation coefficient for the coexpression values of the common 16 110 gene pairs (Figure 3). Expectedly, coexpression data for the same species were well clustered, and species relationships generally obeyed the species tree, so that the primate cluster and rodent cluster appeared in the mammalian cluster. Especially, the platforms for human and those for mouse formed strong clusters, respectively, which are also shown in Figure 2. The high reproducibility suggests high quality of these coexpression data, and was associated with the large number of samples used to construct these coexpression data (Table 1). Note that the KEGG scores for these platforms were not always in the highest levels among all the 23 coexpression platforms (Figure 1, the rightmost bar plot in Figure 3). Among the 11 species, non-mammalian species generally showed higher KEGG scores, implying higher contribution ratio of transcript regulation in pathway regulation in these species.

### Evaluation of each coexpressed gene list

**Figure 4.**
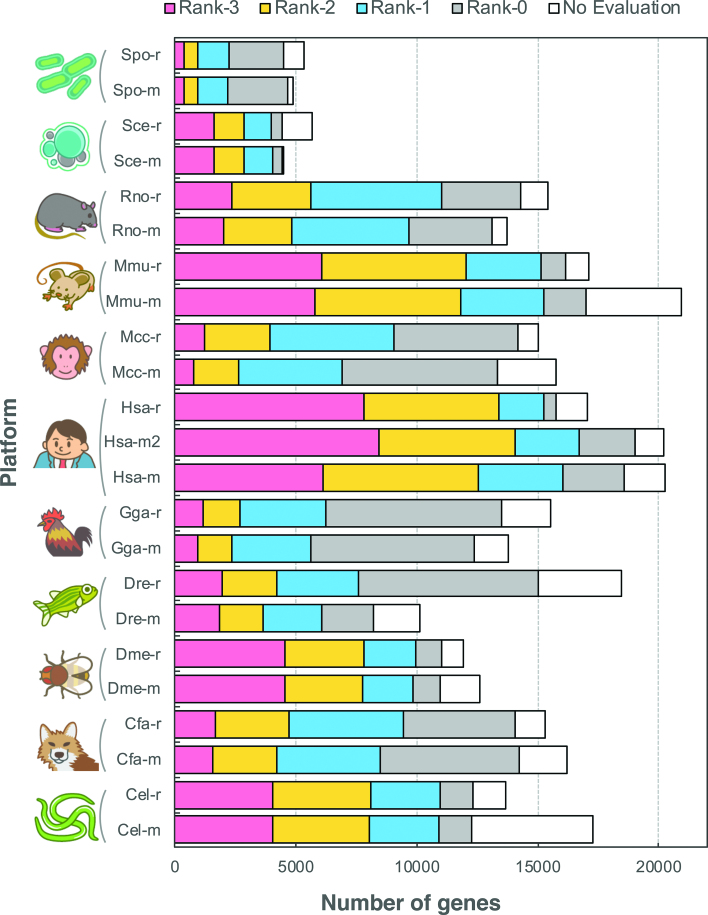
Proportion of supportability level of the coexpressed gene list for every guide gene. As a summarization metric of repeatability of the coexpressed gene list for a guide gene of interest, support was provided, which refers to the similarity of order of the top 1% of the coexpressed gene list with those of the orthologous guide genes. The similarity level is represented as the 4 quantile levels (rank-0 to rank-3) for simplicity.

In the previous section, we focused on the similarity of coexpression platforms to overview their relationships. During actual usage of COXPRESdb, the user often checks coexpressed gene list of a guide gene of interest. In this case, the main concern is reproducibility of individual coexpressed gene list. To provide the reproducibility information of a coexpressed gene list, every coexpression relationship in the gene list has been compared with the identical gene pair in the same species and with the orthologous gene pair in the other species. To visually summarize the degree of coincidence of strong coexpression in the coexpressed gene list, we have introduced *supportability* as described previously (10). For calculation of the supportability, maxCOXSIM_1%_ value is first calculated, which is the maximum weighted coincidence degree of the top 1% genes between two gene lists; a coexpressed gene list of a guide gene of interest and that of the corresponding guide gene in a different coexpression platform. The maxCOXSIM_1%_ value for every coexpressed gene list was then discretized into four quantile levels (0: lowest, 3: highest) for simplicity, shown as zero-star to three-star in the coexpressed gene list of COXPRESdb under the name of *supportability*. Figure 4 shows the proportion of supportability for every coexpression platform. Human and mouse platforms have many rank-3 coexpressed gene lists, which implies that many similar coexpressed gene lists are repeatedly obtained for these species, whereas large proportion of coexpression relationships for monkey (Mcc), chicken (Gga), and fission yeast (Spo) are not well supported by other platforms. Although unreproducible coexpression data is not always false positive relationship, because it depends on quality of the reference coexpression data, it is inconvenient to use such relationship to investigate gene function. The average supportability level for every coexpression platform is shown in Figure 3. The supportability categories quantify coincidence of strongly coexpressed genes (top 1% gene pairs), whereas platform similarity (the matrix in Figure 3) uses any strength of coexpression. Although quantification foci are different, the average supportability and the platform similarity are well consistent. Also, the average supportability and the KEGG scores showed moderate correlation (SCC = 0.58, Figure 3). Although methodologies to assess coexpression data is an on-going challenge, these summaries of coexpression platforms suggest that the current COXPRESdb provides a useful resource of gene coexpression for broad range of species, especially human and mouse (highlighted in Figure 3).

## METHODS

### Preparation of gene expression matrix

Illumina RNAseq entries were downloaded from the DDBJ Sequence Read Archive (16). Based on FASTQ data, quantification of gene expression for the NCBI RefSeq mRNA sequences (17) was performed using Matataki software (18). To reduce uncertainty of measurement of genes with a low expression level, runs including small number of reads were discarded (total mapped counts < 2 000 000). Genes constantly expressed at low levels were omitted (average counts across all runs < 30). After conversion to a base-2 logarithm with a pseudo count of 0.125, batch normalization using ComBat (19) was applied, where an SRP (study) unit was used as a batch unit. Microarray-based transcriptome data were downloaded from ArrayExpress (20). Gene expression values were obtained via RMA method (21) for each downloaded unit, provided as a zip file. Batch normalization was applied using ComBat (19), where a download unit was used as a batch unit. These normalized expression matrices are downloadable in the bulk download page of COXPRESdb [http://coxpresdb.jp/download/].

### Calculation of coexpression data

The construction of coexpression data has been slightly modified from our previous method (2) to retrieve more robust coexpression information. After zero centering of the expression matrix by subtracting the average value for each gene, principal component analysis was applied to obtain independent factors composing gene expression alteration. Weaker principal components (PCs) after the 1000th PC were omitted to reduce calculation cost. To examine the combination of PCs, they were subsampled to be 10% of the number of PCs (i.e. 100 when the number of original samples was >1000). Using the subsampled PCs, coexpression was calculated with Pearson's correlation between any gene pair, and transformed to Mutual Rank by taking geometric average of bi-directional ranks (2,15,22). The procedure of subsampling and coexpression calculation was repeated 1000 times. The 1000 coexpression matrices were averaged in logit-transformed values (2), resulting in a final coexpression matrix for the coexpression platform.
